# Clinical and genetic analysis of a family with cerebrotendinous xanthomatosis

**DOI:** 10.3389/fneur.2025.1566740

**Published:** 2025-05-27

**Authors:** You Guoliang, Wang Zhaoxia, Zhang Lin, Chen Xuan, Zhang Xiaodong, Xing Xiaolian, Su Yangli, Zhang Tianli, Zhu Yanping, Wang Jiangping, Liu Qing

**Affiliations:** ^1^Department of Neurology, Taiyuan City Central Hospital, Taiyuan, China; ^2^Department of Neurology, Peking University First Hospital, Beijing, China; ^3^Department of Neurology, UC Davis School of Medicine, Sacramento, CA, United States

**Keywords:** cerebrotendinous xanthomatosis, CTX, CYP27A1 gene, spastic paraplegia, variant, genetic analysis

## Abstract

**Objective:**

This study aims to analyze the clinical and genetic characteristics of cerebrotendinous xanthomatosis (CTX) in a Chinese family.

**Methods:**

Clinical data, including medical history, neurologic and auxiliary examinations, imaging studies, and genetic profiles were collected from a Chinese CTX family at Taiyuan City Central Hospital. The proband underwent whole exome sequencing, which was confirmed via Sanger sequencing in two affected and five unaffected family members.

**Results:**

Two patients in the pedigree exhibited compound heterozygous missense variants in the *CYP27A1* gene: c.379C > T, (pathogenic variants) and c.397 T > C, (a variant of uncertain clinical significance), both located in exon 2. A literature review revealed that c.1263 + 1G > A and C.379C > T are the most common variants in genetically diagnosed Chinese CTX patients, with exon 2 of the *CYP27A1* gene.

**Conclusion:**

The compound heterozygous variants c.379C > T (p. Arg127Trp) and c.397 T > C (p. Trp133Arg) in the *CYP27A1* gene are likely the cause of CTX in this pedigree. This finding expands our understanding of the genetic and clinical spectrum of CTX and provides significant insights for its diagnosis.

## Background

Cerebrotendinous xanthomatosis (CTX) is an autosomal recessive lipid storage disorder resulting from variants in the *CYP27A1* gene, which encodes the enzyme sterol 27-hydroxylase (1) essential for the bile acid biosynthesis (2). The *CYP27A1* gene is situated on chromosome 2q33-qter and comprises 9 exons and 8 introns. Sterol 27-hydroxylase deficiency leads in excessive accumulation of cholesterol and cholesterol in lipophilic tissues such as tendons, lenses and the brain (3). CTX presents with diverse, multisystem clinical features including chronic diarrhea, juvenile cataracts, tendon xanthomas and neurological disturbances. Over 100 *CYP27A1* mutations have been identified (2), with approximately 50% found in exons 6–8, followed by exon 2 and 4 (4).

In this study, we applied whole exome sequencing to investigate the clinical features and genetic profile of a Chinese pedigree with CTX. Our findings advance the understanding of the disease at both clinical and molecular levels, offering valuable insights for diagnostic and therapeutic applications.

## Methods

### Subjects

In May 2023, a Chinese family consisting of two affected siblings a 29-year-old male proband and a 31-year-old female sibling—along with five unaffected relatives, was recruited from Taiyuan City Central Hospital. Comprehensive clinical data, including clinical manifestations, imaging studies, assessments, and pathological examinations, were collected. Whole exome sequencing was conducted for the proband, and Sanger sequencing was used to validate the genetic variants in both affected siblings and the five unaffected family members. Written informed consent for publication and ethical approval for the study were obtained from all participants, as approved by the Research Ethics Committee of Taiyuan City Central Hospital.

### Whole exome sequencing

Whole exome sequencing was performed on the proband. Genomic DNA was extracted from peripheral blood leukocytes, and DNA libraries were constructed using the KAPA Library Preparation Kit (Kapa Biosystems, United States). The library preparation included three standard steps: DNA fragmentation end-repair, A-tailing, and adapter ligation and amplification. Libraries concentrations were quantified using the Qu bit dsDNA HS Assay kit (Invitrogen, United States). Sequencing was carried out on the Illumina NovaSeq platform (Illumina United States) using paired-end 200-bp reads.

### Sanger sequencing

Sanger sequencing was used to validate the genetic variants in both affected patients and five unaffected family members. Genomic DNA was extracted from blood samples using a blood extraction kit (Beijing Baitech Biotechnology, China). Specific primers were designed to amplify the DNA fragments covering the variant site. The PCR products were sequenced using an ABI 3730XL DNA Analyzer (Applied Biosystems, United States).

### Literature review

An extensive literature review was conducted using the PubMed database with the keywords “Cerebrotendinous Xanthomatosis” and “China.” The search was last updated on November 8th, 2024. Only full-text articles published in English were included in the review.

## Results

### Clinical characterization

**Figure 1:**
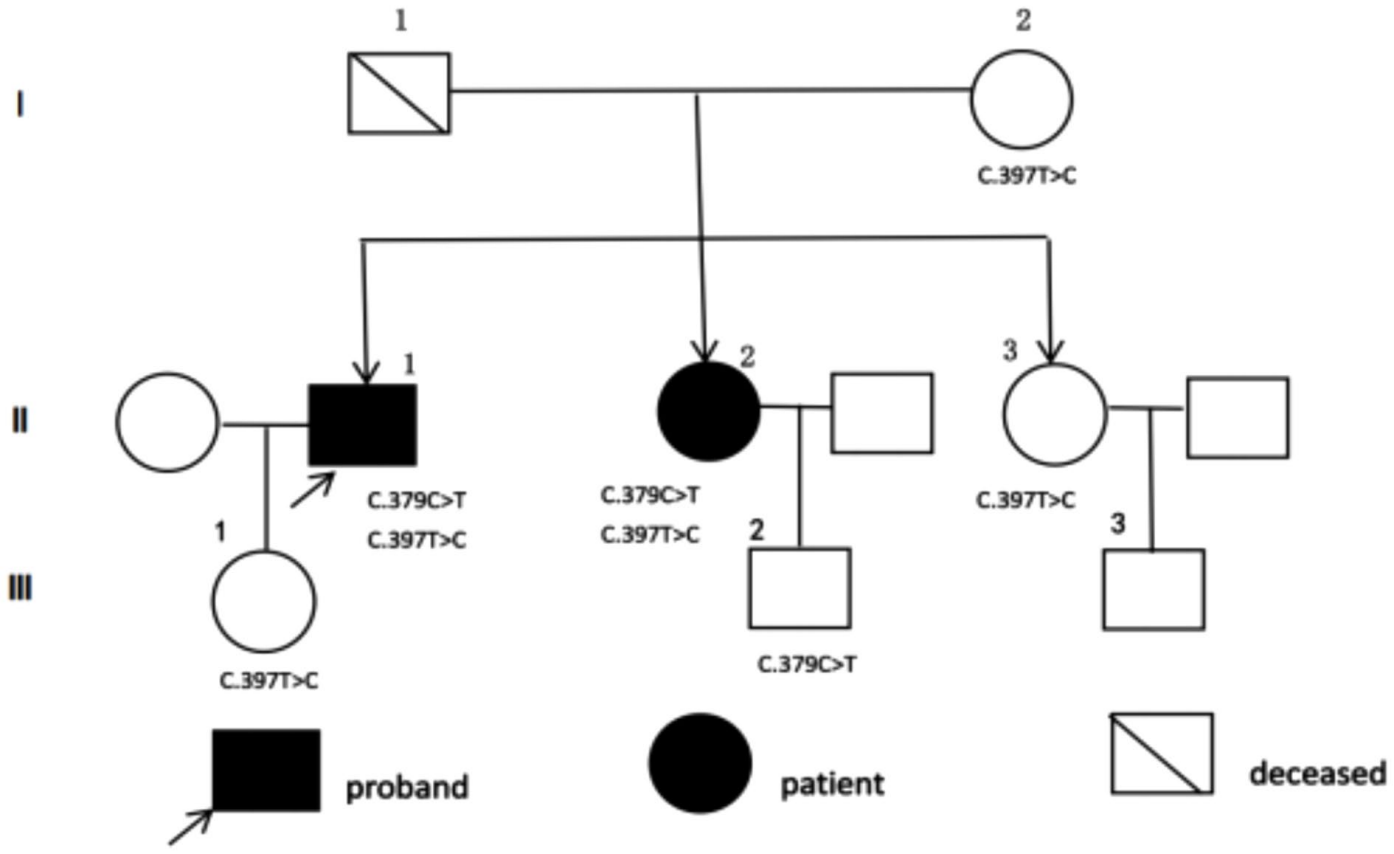
The pedigree diagram of this CTX family.

Figure 1 illustrates the pedigree diagram of this CTX family.

#### Case 1

**Figure 2:**
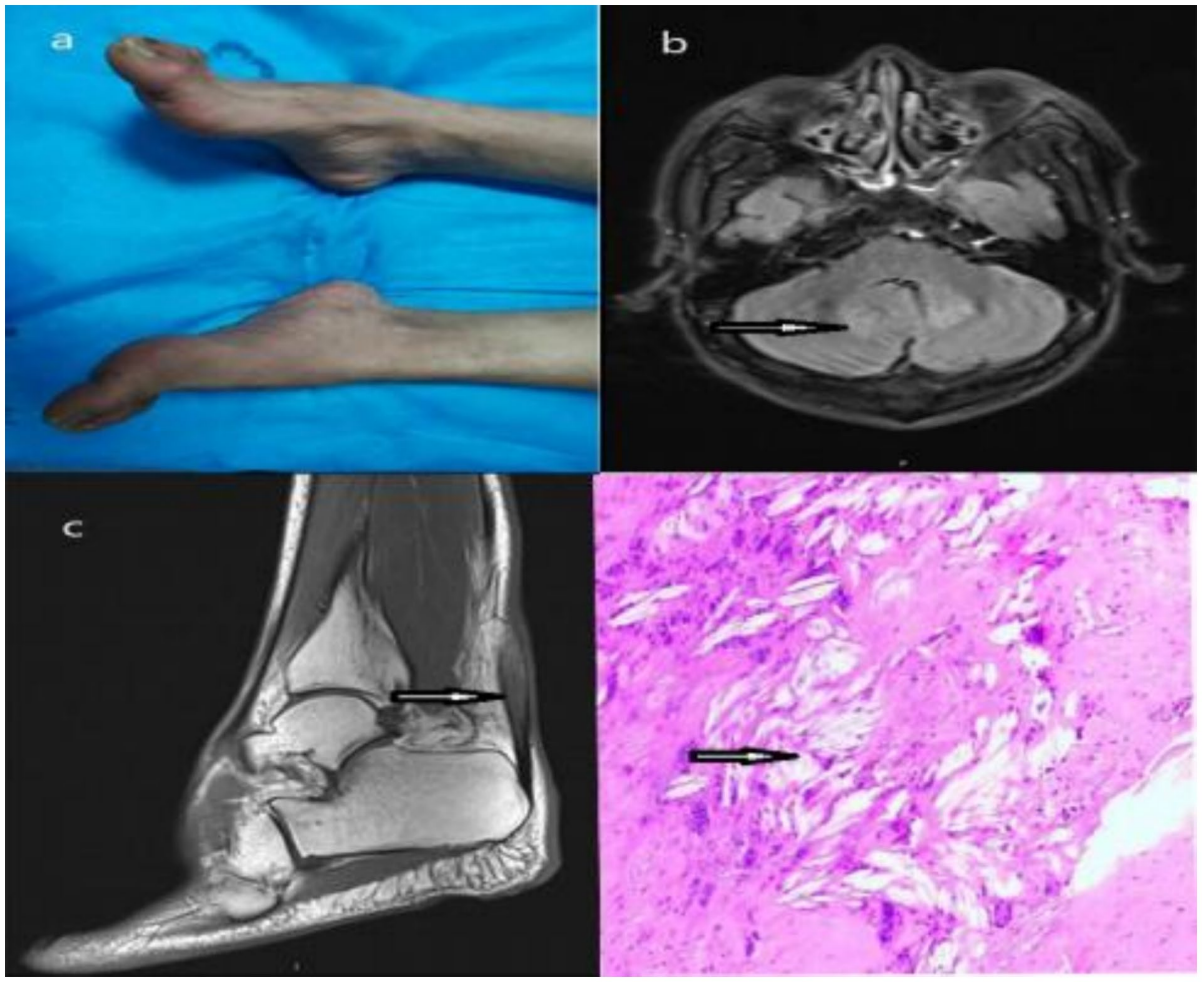
**(a)** The proband exhibits pes cavus deformity in both feet. **(b)** Brain MRI findings demonstrated symmetric hyperintensity in bilateral cerebellar dentate nuclei on FLAIR images (arrow). **(c)** MRI of Achilles tendon showed slight hyperintensity in Achilles tendon on FLAIR images (arrow). **(d)** Postoperative pathological analysis of the proband’s second elder sister’s Achilles tendon mass showed a significant presence of cholesterol crystals (HE × 100; arrow).

The proband (II1), a 29-year-old male patient was admitted to our hospital with chronic diarrhea, bilateral cataracts, spastic paraplegia, and cognitive impairment. He had experienced chronic diarrhea since age of 8 and was diagnosed with bilateral cataracts at age 10. At age 27, he developed weakness in both lower extremities and unsteady gait. At age 28, he began having frequent falls but was still able to walk independently. A physical examination revealed bilateral cataracts, but no significant tendon xanthomas were noted on the Achilles tendons. Neurological examination showed reduced visual acuity in both eyes. His cognitive abilities were notably impaired, particularly in calculation and comprehension, with a Mini-Mental State Examination (MMSE) score of 17 and a MoCA score of 10. He demonstrated a paraparetic gait, bilateral hyperreflexia of the knee tendons, positive Babinski signs bilaterally, and positive patella and ankle clonus. Muscle strength of the lower limbs was rated 5−/5. Additionally, pes cavus deformity was observed in both feet (Figure 2a).

Laboratory examinations revealed a normal serum cholesterol level. Magnetic resonance imaging (MRI) of the brain showed symmetric hyperintensity in the bilateral cerebellar dentate nuclei on fluid-attenuated inversion recovery (FLAIR) images (Figure 2b). MRI of the Achilles tendon demonstrated slight hyperintensity in the Achilles tendon on FLAIR images (Figure 2c). Nerve conduction studies showed reduced motor conduction velocity and amplitude in both the tibia l and common peroneal nerves.

#### Case 2

The proband’s 31-year-old female older sister presented without neurological symptoms but had cataracts and masses on her Achilles tendons. She underwent cataract surgery during her adolescence and had a surgical procedure in 2020 to remove the masses from her Achilles tendons. Postoperative pathological analysis of the Achilles tendon mass, stained with hematoxylin and eosin staining (HE), revealed the presence of cholesterol crystals (Figure 2d). Despite the absence of reported neurological symptoms, her neurological examination showed positive clinical signs, including a paraparetic gait, bilateral hyperreflexia of knee tendons, bilateral positive Babinski signs and pes cavus deformity in both feet.

### Genetic analysis

**Figure 3:**
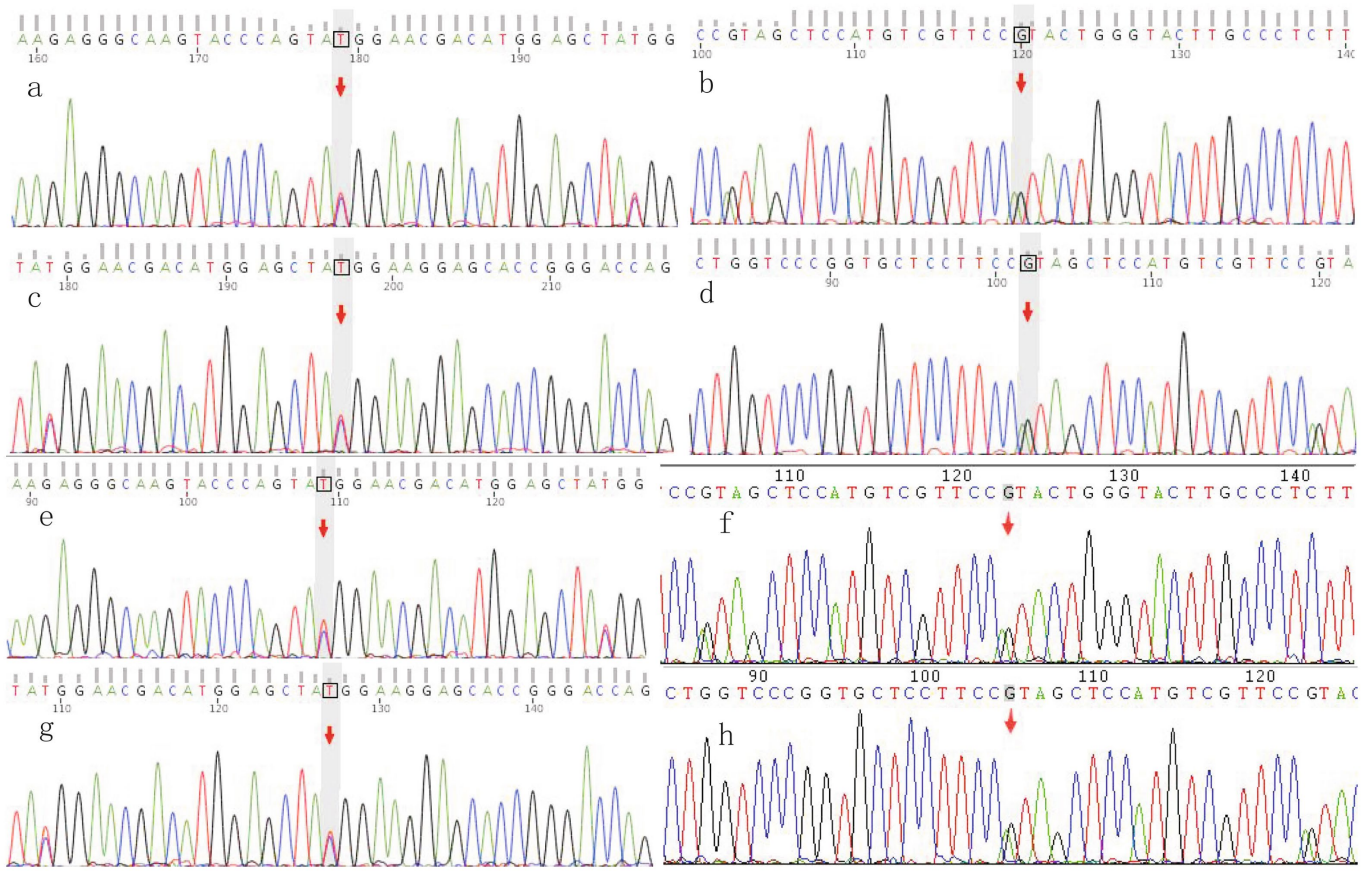
Sanger sequencing analysis of two cases. **(a,b)** Sanger sequencing results for the proband demonstrating c.379C > T variant on CYP27A1 gene (**a**. forward; **b**. reverse). **(c,d)** Sanger sequencing results for the proband demonstrating c.397 T > C variant on CYP27A1 gene (**c**. forward; **d**. reverse). **(e,f)** Sanger sequencing results for the proband’s second elder sister demonstrating c.379C > T variant on CYP27A1 gene (**e**. forward; **f**. reverse). **(g,h)** Sanger sequencing results for the proband’s second elder sister demonstrating c.397 T > C variant on CYP27A1 gene (**g**. forward; **h**. reverse).

Whole exome sequencing complemented by Sanger sequencing, revealed identical compound heterozygous variants, c.379C > T and c.397 T > C, in exon 2 of the CYP27A1 gene for both affected individuals (Figure 3). Sanger sequencing analysis of five unaffected family members revealed that individuals I2, II3 and III1 are carriers of c.397 T > C variant, while individual III2 is a carrier of c.379C > T variant; neither mutation was detected in individual III3. The c.397 T > C was inherited from the mother. Blood samples were unavailable from the father due to his passing but based on the shared variants in both parents. We hypothesized that the c.379C > T variant originated from the father. The genetic findings for this family are summarized in Table 1.

**Table 1:** The results of the genetic analysis in this family.

Family members	C.379C > T	C.397 T > C
I2	Negative	Positive
II1	Positive	Positive
II2	Positive	Positive
II3	Negative	Positive
III1	Negative	Positive
III2	Positive	Negative
III3	Negative	Negative

According to the American College of Medical Genetics (ACMG) and the Association for Molecular Pathology (AMP) guidelines, c.379C > T variant was classified as pathogenic (PM3_PS + PM2 + PP3 + PS3_PP) and c.397 T > C variant was classified as VUS (PM2 + PP3). In-silico analysis by REVEL of these two variants reveals high impact, c.379C > T variant got a REVEL score 0.653 and c.397 T > C variant got a REVEL score 0.84.

### Literature review

A total of 32 eligible Chinese patients from 28 families with CTX were identified from 14 studies. The primary clinical manifestations, neuroimaging findings, and genetic characteristics of these patients were summarized in Table 2. Of these patients, 20 (62.5%) were male and 12 (37.5%) were female. The average age of presentation was 15.7 years (standard deviation, SD = 13.7), ranging from 1 to 42 years. The most common neurological symptoms were pyramidal signs (27, 84.4%), ataxia (20, 62.5%) and cognitive decline (19, 59.4%). In contrast, psychiatric disturbances (9, 28.1%), peripheral neuropathy (8, 25%), and movement disorders (4, 12.5%) were less frequently reported. Among systemic hallmark symptoms, tendon xanthomas were common (23, 71.9%) and cataract (14, 43.8%) were frequent, while chronic diarrhea (11, 34.4%) was relatively rare. Results of neuroimaging were not available for 4 patients, and 22(68.8%) of the remaining patients exhibited dentate nuclei signal alteration at brain MRI. Genetic analysis was performed in all cases except one, revealing 2 homozygous mutations and 29 compound heterozygous variants. The most prevalent variants included c.1263 + 1G > A, c.379C > T, c.435G > T, and c.1420C > T.

**Table 2:** Clinical, neuroimaging and molecular genetic features in previously reported Chinese patients with CTX.

Case	Author/at symptom on set	Gender	Age at onsetly	Neurological symptom						Cataracts	Tendon xanthoma	Diarrhea	Dentate nuclei signal alteration At MRI	Genetic mutation
				Ataxia	pyramidal signs	Psychiatric disturbances	Movement disorders	Cognitive decline	Peripheral neuropathy					
1	Chi Ma et al., 2021 (12)	M	38	−	+	−	−	+	−	−	+	−	+	Not Applicable
2	Qing-Qing Tao et al., 2019 (13)	M	36	+	+	−	−	−	+	−	+	−	+	compound heterozygous variant c.571C > T c.435G > T
3	Qing-Qing Tao et al., 2019 (13)	M	37	+	+	−	−	−	−	−	+	−	+	compound heterozygous variant c.1214G > A c.1435C > T
4	Qing-Qing Tao et al., 2019 (13)	M	6	−	+	−	−	+	−	+	+	−	+	homozygous variant c.1435C > T
5	Qing-Qing Tao et al., 2019 (13)	M	12	+	+	+	−	−	−	−	−	−	Not Applicable	compound heterozygous variant c.368_374delCCAGTAC c.379C > T
6	Qing-Qing Tao et al., 2016 (13)	M	4	−	+	−	−	+	−	−	−	−	+	compound heterozygous variant c.1016C > T c.1420C > T
7	Qing-Qing Tao et al., 2016 (13)	M	26	−	+	−	−	−	−	−	−	−	Not Applicable	compound heterozygous variant c.389 T > A c.1263 + 1G > A
8	Shu Zhang et al., 2020 (14)	F	2	+	+	+	−	+	+	+	+	+	+	compound heterozygous variant c.1263 + 1G > A c.1420C > T
9	Shu Zhang et al., 2020 (14)	M	25	−	−	−	−	+	+	−	+	−	+	compound heterozygous variant c.472C > T c.432 T > G
10	Shu Zhang et al., 2020 (14)	M	2	+	+	−	−	+	+	+	−	+	+	compound heterozygous variant c.1263 + 1G > A c.1055C > A
11	Shu Zhang et al., 2020 (14)	M	2	+	+	−	−	+	+	+	−	+	+	compound heterozygous variant c.1263 + 1G > A c.1004C > T
12	Shu Zhang et al., 2020 (14)	M	2	+	+	−	−	+	+	+	−	+	+	compound heterozygous variant c.1214G > A c.1004C > T
13	Shu Zhang et al., 2020 (14)	M	3	+	+	−	−	+	+	+	+	−	+	compound heterozygous variant c.379\u00B0C > T c.1263 + 1G > A
14	Chen Chen et al., 2017 (15)	M	33	+	+	−	−	−	−	−	+	−	−	compound heterozygous variant c.1477-2A > C c.1537\u00B0C > T
15	Chen Chen et al., 2017 (15)	F	30	−	−	−	−	−	−	−	+	−	Not Applicable	compound heterozygous variant c.1477-2A > C c.1537\u00B0C > T
16	Chen Chen et al., 2017 (15)	M	33	−	+	−	−	−	−	−	+	−	−	homozygous variant c.410G > A
17	Chen Chen et al., 2017 (15)	F	42	−	−	−	−	−	−	−	+	−	−	compound heterozygous variant c.410G > A c.379C > T
18	Chen Chen et al., 2017 (15)	F	33	+	+	−	−	+	−	−	+	−	+	compound heterozygous variant c.562C > T c.1214G > A
19	Lan-Xiao Cao et al., 2017 (16)	M	17	−	−	+	−	+	−	−	+	−	−	compound heterozygous variant c.435G > T c.1263 + 1G > A
20	Chen-Xi Ling et al., 2023 (17)	F	33	+	+	+	−	−	−	+	+	−	+	compound heterozygous variant c.379C > T c.1263 + 3G > C
21	Yue-Yue Chang et al., 2022 (18)	F	9	+	+	+	+	−	−	+	+	−	+	compound heterozygous variant c.255 + 1G > T c.1263 + 1G > T
22	Zhao-Ran Li et al., 2022 (19)	M	8	+	+	+	−	+	−	−	+	−	+	compound heterozygous variant c.380G > A c.1563dupA
23	Jing-wen Jiang et al., 2020 (20)	M	7	+	+	−	−	+	−	−	−	+	−	compound heterozygous variant c.1263 1G > A c.1561dupA
24	Jing-wen Jiang et al., 2020 (20)	F	8	−	+	−	−	−	−		−	+	+	compound heterozygous variant c.1263 1G > A c.1537C > T
25	Jing-wen Jiang et al., 2020 (20)	F	9	+	+	+	−	+	−	+	+	−	+	compound heterozygous variant c.255 1G > T c.1263 1G > A
26	Jing-wen Jiang et al., 2020 (20)	M	14	+	+	+	−	−	−	+	−	+	+	compound heterozygous variant c.379C > T c.1263 1G > A
27	Zhao-xia Wang et al., 2007 (21)	F	6	−	+	−	−	+	+	+	+	−	+	compound heterozygous variant c.379C > T c.1420C > T
28	Jun Li et al., 2019 (22)	M	9	+	+	−	+	−	−	+	+	+	+	compound heterozygous variant c.1016C > T c.1263 + 1G > A
29	Yi Tang et al., 2020 (23)	F	1	+	+	+	+	+	−	+	+	+	+	compound heterozygous variant c.435G > T c.562C > T
30	Yi Tang et al., 2020 (23)	M	2	+	−	−	−	+	−	−	+	+	Not Applicable	compound heterozygous variant c.435G > T c.562C > T
31	Di Tian et al., 2011 (24)	F	7	−	+	−	−	+	−	+	+	+	+	compound heterozygous variant c.73delG c.369_375delGTACCCA
32	Wei Zhao et al., 2020 (25)	F	7	+	+	−	+	+	−	−	+	−	−	compound heterozygous variant c. 1214G > A c. 1420C > T

We have compiled literature on cerebrotendinous xanthomatosis from Turkey, Brazil, Spain, and France, encompassing a total of 178 reported cases. Among these cases, the common neurological symptoms include pyramidal signs (102, 57.3%), ataxia (120, 67.4%), cognitive decline (126, 71.3%), psychiatric disturbances (111, 62.4%), peripheral neuropathy (64, 51.2%).While the common non-neurological symptoms are cataract (138, 77.5%), chronic diarrhea (88, 49.4%), tendon xanthomas (110, 61.8%) (5–8). The most prevalent variants observed in: Turkish CTX patients: c.476 + 27C > T, c.808C > T, C.1263 + 4A > T, Brazilian CTX patients: c.1183C > T, c.379C > T, French CTX patients: c.1183C > T, c.1184 + 1G > A, Spanish CTX patients: c.1183C > T, C.1213C > T (5–8).

## Discussion

CTX is an autosomal recessive disorder caused by defects in bile acid biosynthesis, resulting in the accumulation of cholesterol and cholesterol in various tissues including the central nervous system, tendons, and lenses. The condition typically has a gradual progression, presenting a broad spectrum of neurological and non-neurological manifestations.

In our review of Chinese CTX cases, tendon xanthoma and cataracts were the most common non-neurological symptoms, while chronic diarrhea was less frequently observed. The typical clinical progression of CTX in China begins with cataracts in childhood, followed by the appearance of tendon xanthomas in adolescence, with neurological symptoms—such as ataxia, dementia, peripheral neuropathy, dystonia, spastic paraplegia, and epilepsy—emerging in adulthood.

Comparative analysis reveals distinct phenotypic profiles between Chinese and international CTX cases. Beyond the shared ono-neurological manifestations of cataracts and tendon xanthomas, chronic diarrhea emerges as a hallmark feature in global cohorts. Neurologically, while pyramidal signs, ataxia, and cognitive decline constitute the core symptomatology worldwide, psychiatric disturbances and peripheral neuropathy are notably prevalent in non-Chinese populations. The common genetic variants in Chinese CTX patients are notably distinct from those observed in the aforementioned four countries, while significant inter-varietal disparities also exist among these four nations, suggesting important regional differences in CTX genetic profiles.

In this family, the clinical presentation mirrored the typical Chinese CTX progression. Non-neurological signs included cataracts, pes cavus, chronic diarrhea, while the primary neurological symptoms were peripheral neuropathy, cognitive dysfunction, and spastic paraplegia. This pattern aligns with the broader literature, which shows that neurological manifestations often follow non-neurological signs.

These findings emphasize the importance of recognizing early non-neurological signs, such as cataracts and tendon xanthomas, to enable timely diagnosis and early intervention in CTX cases.

Despite CTX being classified as a rare disorder, it is frequently underdiagnosed due to its highly variable clinical presentation and the extensive spectrum of the disease. To date, hundreds of cases have been described, yet the exact number of undiagnosed or potentially misdiagnosed instances remains uncertain (9). In this family the proband did not display the typical tendon xanthomas; but instead presented with spastic paraplegia and ataxia, complicating the diagnostic process. However, their history of chronic diarrhea and juvenile-onset cataracts raised suspicion for CTX. This case underscores the importance of considering CTX in patients with chronic diarrhea, particularly when early cataracts are present.

It is also apparent that many non-neurologists may not have the familiarity of CTX. In case 2, the patient underwent surgical excision of an Achilles tendon mass in the orthopedic department, and the pathological analysis revealed cholesterol crystals. However, the diagnosis of CTX had not yet been established. This highlights the need for increased awareness among healthcare professionals, particularly those outside neurology, to consider CTX in patients presenting with characteristic clinical features such as tendon messes, cataracts, and chronic diarrhea. Early recognition and genetic testing are essential for confirming the diagnosis and preventing further disease progression.

The **CYP27A1** gene, located on chromosome 2q35, spans 9 exons and 8 introns, encoding a total of 531 amino acids. Pathogenic variants associated with CTX are spread across all exons and several introns (2, 4, 6, 7, and 8), with nearly half occurring within exons 6–8 (4). In this study, we report a compound heterozygous variant in the **CYP27A1** gene in a Chinese family with two CTX patients, specifically the c.379C > T and c.397 T > C variants, both located in exon 2.

The **c.379C > T** variant, located at base 219,674,423 on chromosome 2 involves the substitution of cytosine (C) with thymine (T) at the 379th position. This leads to the replacement of the 127th encoded amino acid from arginine (Arg) to tryptophan (Trp). This variant has been reported in multiple CTX patients, and *in vitro* analyses have shown that it disrupts protein function, supporting its classification as pathogenic.

The **c.397 T > C** mutation, located at the position 219,674,441 on chromosome 2, involves the substitution of thymine (T) with cytosine (C) at position 397th, resulting in the change of the 133rd amino acid from tryptophan (Trp) to arginine (Arg). This variant was inherited maternally and has been reported in only two cases (10, 11), with no familial instances documented. Based on available data, it is classified as having uncertain clinical significance.

## Conclusion

The identification of the compound heterozygous mutation **c.379C > T** (p. Arg127Trp) and **c.397 T > C** (p. Trp133Arg) in the **CYP27A1** gene is likely responsible for the occurrence of CTX. This discovery enhances our understanding of the genetic and clinical spectrum of CTX and provides valuable insights for its diagnosis and clinical management.

## Data Availability

The datasets presented in this study can be found in online repositories. The names of the repository/repositories and accession number(s) can be found in the article/supplementary material.
